# High Glucose Induces Podocyte Injury via Enhanced (Pro)renin Receptor-Wnt-β-Catenin-Snail Signaling Pathway

**DOI:** 10.1371/journal.pone.0089233

**Published:** 2014-02-12

**Authors:** Caixia Li, Helmy M. Siragy

**Affiliations:** Division of Endocrinology and Metabolism, University of Virginia Health System, Charlottesville, Virginia, United States of America; Max-Delbrück Center for Molecular Medicine (MDC), Germany

## Abstract

(Pro)renin receptor (PRR) expression is upregulated in diabetes. We hypothesized that PRR contributes to podocyte injury via activation of Wnt-β-catenin-snail signaling pathway. Mouse podocytes were cultured in normal (5 mM) or high (25 mM) D-glucose for 3 days. Compared to normal glucose, high glucose significantly decreased mRNA and protein expressions of podocin and nephrin, and increased mRNA and protein expressions of PRR, Wnt3a, β-catenin, and snail, respectively. Confocal microscopy studies showed significant reduction in expression and reorganization of podocyte cytoskeleton protein, F-actin, in response to high glucose. Transwell functional permeability studies demonstrated significant increase in albumin flux through podocytes monolayer with high glucose. Cells treated with high glucose and PRR siRNA demonstrated significantly attenuated mRNA and protein expressions of PRR, Wnt3a, β-catenin, and snail; enhanced expressions of podocin mRNA and protein, improved expression and reorganization of F-actin, and reduced transwell albumin flux. We conclude that high glucose induces podocyte injury via PRR-Wnt- β-catenin- snail signaling pathway.

## Introduction

High glucose contributes to glomerular injury and a progressive renal function loss, leading to end-stage renal disease (ESRD)[1], [2]. Podocytes are important component of the glomerular basement membrane and involved in several key functions, mainly limiting albumin filtration [3]. Podocyte injury is characterized by decreased expression of slit diaphragm-associated proteins, nephrin and podocin and increased albumin filtration [4], [5]. Previous studies identified podocyte injury as a key early event leading to glomerular disease [6], seen in patients with diabetic nephropathy [7], [8]. However, the mechanisms involved in high glucose induced podocyte injury are not well established.

In the kidney, hyperglycemia activates all components of the renin-angiotensin system (RAS) [9], [10], contributing to the development of diabetic nephropathy. However, despite the utilization of RAS inhibitors, some patients with this disease continue to progress to ESRD [11], [12].

The (pro)renin receptor (PRR) is a 350-amino acid protein with four different domains: an N-terminal signal peptide, an extracellular domain, a signal transmembrane domain and a short cytoplasmic domain [13], [14], [15]. PRR is expressed in the kidney, mainly in the glomerular mesangial cells [16], vascular smooth muscle cells [13], proximal and distal renal tubules [17], and podocytes [18]. Recently we reported that PRR is up-regulated in the kidneys of diabetic rats [19] and in mesangial cells exposed to high glucose. Activation of PRR generates intracellular signal molecules, such as phosphorylation ERK1/2 and p38, leading to inflammation and matrix formation [16], [18], [20], [21], [22]. Down-regulation of PRR expression reversed high glucose induced inflammation [16], [23], implying that PRR may contribute to the pathophysiology of diabetic kidney disease. However, it is not clear how PRR contributes to renal injury induced by hyperglycemia.

The Wnt gene encodes a large family of secreted proteins that have been identified from Hydra to Human [24], [25], [26]. Wnts are involved in functions governing cell fate, proliferation, migration, polarity and death [27], [28], [29] through at least three distinct intracellular pathways, including the canonical Wntβ-catenin signaling pathway, the non-canonical Wnt-Ca2+ pathway, and Wnt-PCP (Planar Cell Polarity) pathway [24], [30], [31]. Wnt-β-catenin pathway is involved in several developmental and pathologic processes including cancer [32], [33], fibrosis[34], [35], cystic disease [36], renal failure [37], and diabetic nephropathy [38]. Canonical Wnt-β-catenin signaling pathway expression is increased in glomeruli and podocytes of hyperglycemic patients and mouse model of diabetic kidney disease and plays a critical role in integrating cell adhesion, motility, cell death, and differentiation [38]. Recently, PRR was found to be an accessory subunit for vacuolar (V-ATPase), which contributes to the activation of the canonical Wnt-β-catenin signaling pathway [39]. However, it is unknown whether the PRR induced canonical Wnt-β-catenin signal activation occurs and contributes to high glucose-induced podocytes injury.

In this study, we investigated the role of enhanced PRR expression in high glucose-induced podocyte injury. Our results demonstrated that high glucose-induced podocyte structure and function changes are mediated by up regulation of PRR via activation of the canonical Wnt3a-β-catein-snail signaling pathway.

## Results

### PRR mRNA and protein expression

**Figure 1:**
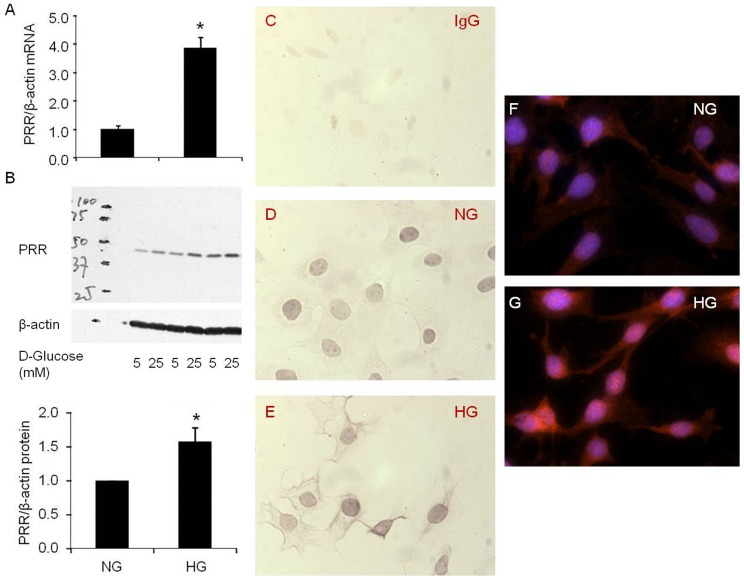
Effect of high glucose on PRR expression in podocytes. A. Real time PCR analysis of PRR mRNA expression in response to high glucose for 72(n = 6); B. Western blot analysis of PRR protein expression in response to high glucose for 72 hours (n = 6); C, D and E. Immunohistochemistry staining of PRR shown in brown (n = 3); F and G. Immunofluorescence staining of PRR shown in red, DAPI shown in blue (n = 5).PRR, (Pro)renin receptor; normal glucose, 5 mM D-glucose (NG); high glucose, 25 mM D-glucose (HG). Data presented as mean ± SEM, *p<0.05 *vs* NG

Compared to normal glucose, high glucose significantly increased expression of PRR mRNA by 285% (Fig 1A, p<0.001) and protein by 57% (Fig 1B, p<0.05). Similarly, high glucose treatment significantly increased PRR immunostaining (Fig. 1D, 1E, 1F and 1G).

### Podocin and nephrin mRNA and protein expressions, F-actin immunostaining and functional monolayer permeability

**Figure 2:**
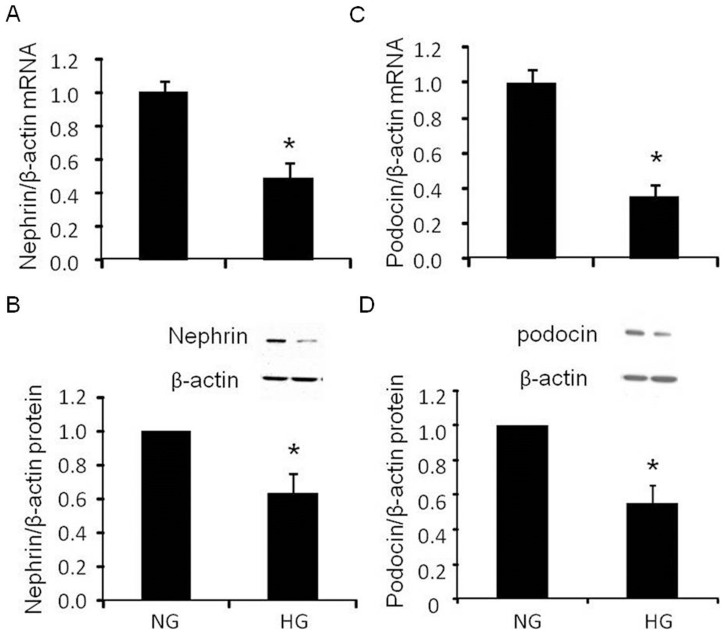
Effect of high glucose on nephrin and podocin expression in podocytes. A. Real time PCR analysis of nephrin mRNA expression (n = 5); B. Western blot analysis of nephrin protein expression (n = 6); C. Real time PCR analysis of Podocin mRNA expression (n = 5); D. Western blot analysis of Podocin protein expression (n = 4). Normal glucose, 5 mM D-glucose (NG); high glucose, 25 mM D-glucose (HG). Data presented as mean ± SEM, *p<0.05 *vs* NG

**Figure 3:**
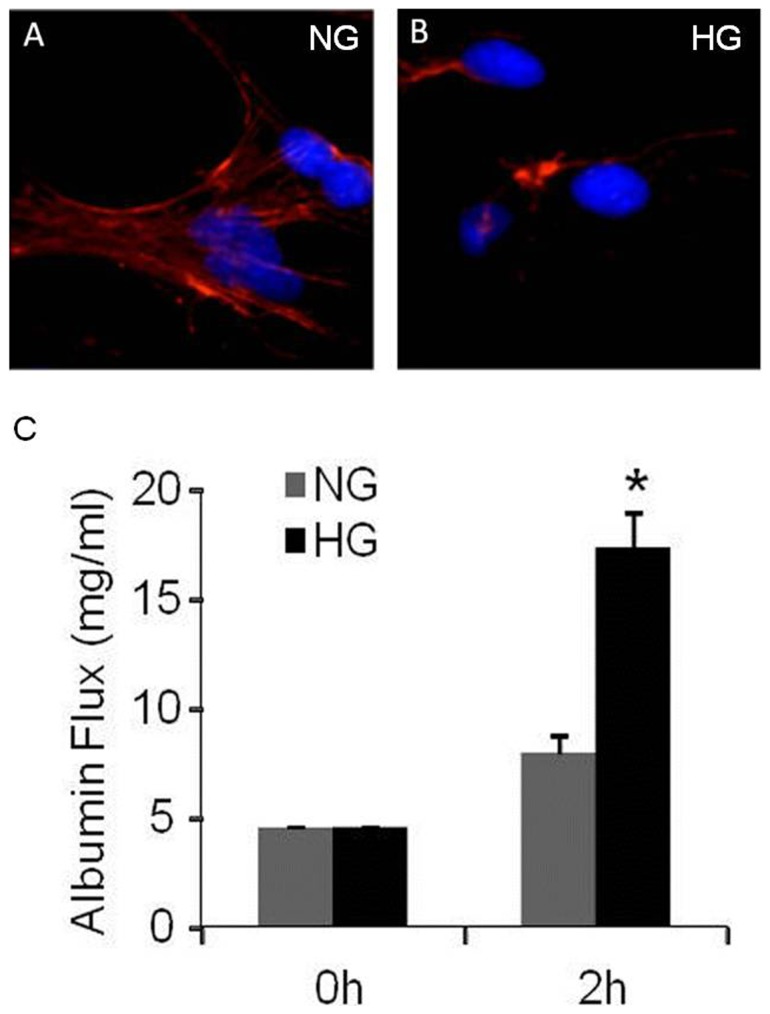
Effect of high glucose on structure and function injury in podocytes. A and B Immunofluorescence staining of F-actin showed in red, DAPI showed in blue (n = 5); C. Analysis of the albumin flux across podocytes monolayer (n = 5). Normal glucose, 5 mM D-glucose (NG); high glucose, 25 mM D-glucose (HG). Data presented as mean ± SEM, *p<0.05 *vs* NG

High glucose significantly reduced mRNA and protein levels of nephrin (Fig 2A, p<0.01 and 2B, p<0.05) and podocin (Fig 2C, p<0.001 and 2D, p<0.05). Similarly, high glucose significantly reduced immunostaining of podocyte cytoskeleton F-actin protein expression and induced its disorganization (Fig 3A and B). Functional podocyte permeability assay showed significant increase in albumin flux in high glucose treated cells at 2 hours (Fig 3C, 8 vs. 17 mg/ml, p<0.001).

### Wnt3a, β-catenin and snail mRNA and protein expressions

**Figure 4:**
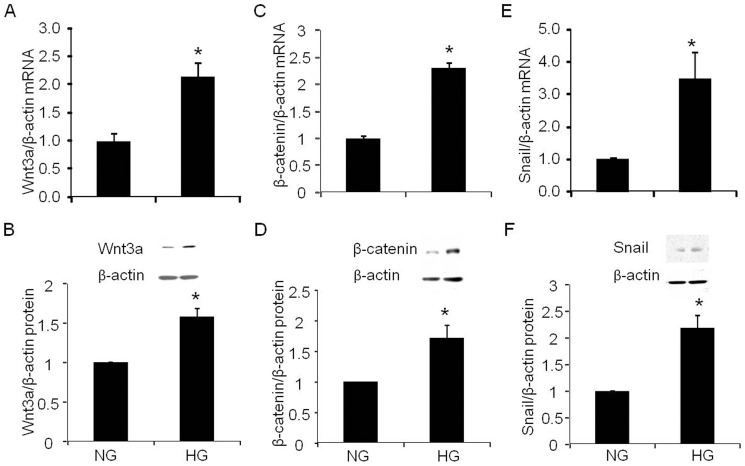
Effect of high glucose on Wnt3a-β-catenin-snail signal pathway in podocytes. A. Real time PCR analysis of Wnt3a mRNA expression (n = 5); B. Western blot analysis of Wnt3a protein expression (n = 4); C. Real time PCR analysis of β-catenin mRNA expression (n = 6); D. Western blot analysis of β-catenin mRNA expression (n = 5); E. Real time PCR analysis of snail mRNA expression (n = 5); F. Western blot analysis of snail protein expression (n = 4); Normal glucose, 5 mM D-glucose (NG); high glucose, 25 mM D-glucose (HG). Data presented as mean ± SEM, *p<0.05 *vs* NG

Compared to normal glucose, high glucose significantly increased mRNA and protein expressions of Wnt3a (Fig 4A, 114%, p<0.05; Fig 4B, 38%, p<0.01), β-catenin (Fig 4C, 129%, p<0.001; Fig 4D, 71%, p<0.05) and snail (Fig 4E, 250%, p<0.05; Fig4F, 101%, p<0.05), respectively.

### PRR expression in PRR siRNA transfected podocytes

**Figure 5:**
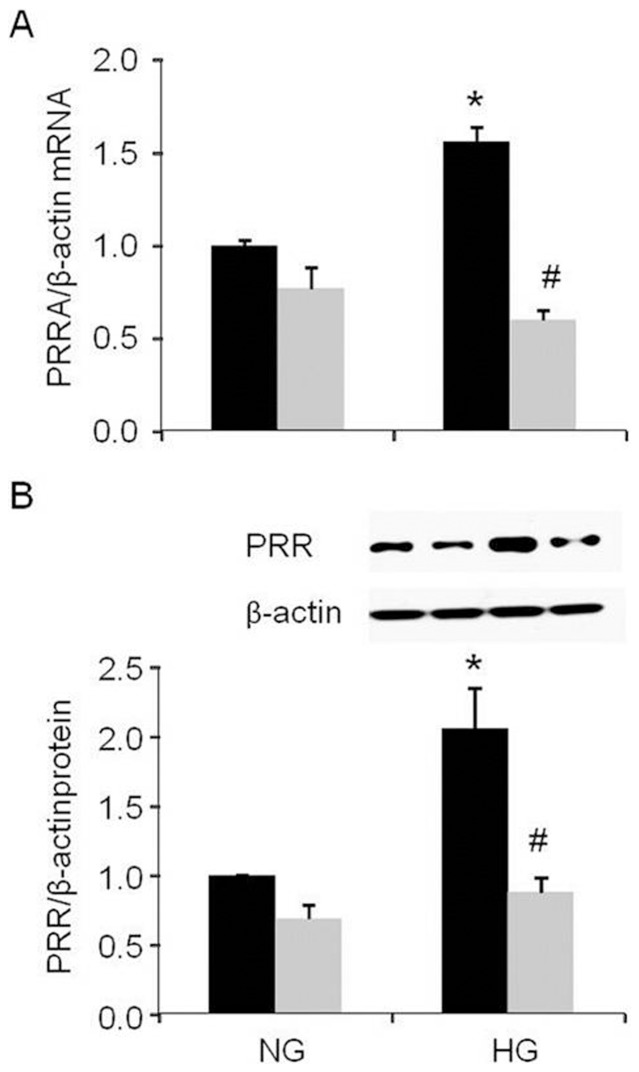
Effect of PRR siRNA on PRR expression in podocyte upon high glucose. A. Real time PCR analysis of PRR mRNA expression (n = 6); B. Western blot analysis of PRR protein expression (n = 5–6); PRR, (Pro)renin receptor; Normal glucose, 5 mM D-glucose (NG); high glucose, 25 mM D-glucose (HG). Black bar, Scrambled siRNA; Grey bar, PRR siRNA. Data presented as mean ± SEM, *p<0.05 *vs* NG+ Scrambled siRNA; #*p*<0.05 *vs* HG+ Scrambled siRNA.

High glucose significantly increased the expression of PRR mRNA by 56% and protein by 106% (Fig 5A and B). PRR siRNA treatment significantly attenuated expression of PRR mRNA by 62% (Fig 5A, p<0.001) and protein by 58% (Fig 5B, p<0.05) in high glucose treated cells.

### Wnt3a, β-catenin and snail expression in PRR siRNA transfected podocytes

**Figure 6:**
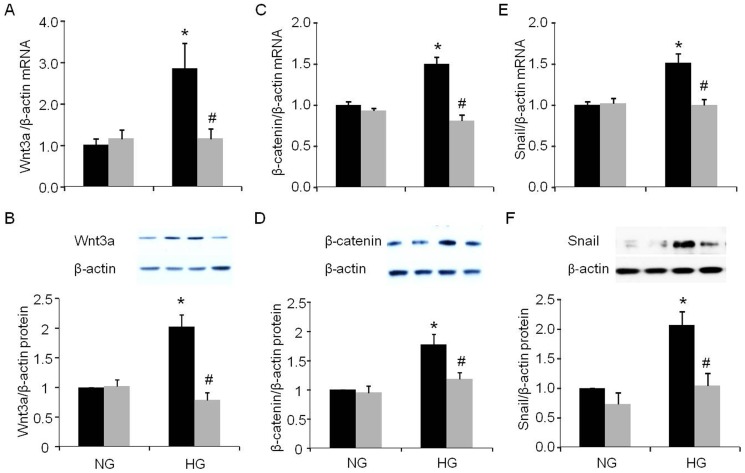
Effect of PRR siRNA on Wnt3a-β-catenin-snail signal pathway in podocytes in response to high glucose. A. Real time PCR analysis of Wnt3a mRNA expression (n = 5); B. Western blot analysis of Wnt3a protein expression (n = 6); C. Real time PCR analysis of β-catenin mRNA expression (n = 6); D. Western blot analysis of β-catenin mRNA expression (n = 6); E. Real time PCR analysis of snail mRNA expression (n = 5); F. Western blot analysis of snail protein expression (n = 4); PRR, (Pro)renin receptor; Normal glucose, 5 mM D-glucose (NG); high glucose, 25 mM D-glucose (HG). Black bar, Scrambled siRNA; Grey bar, PRR siRNA. Data presented as mean ± SEM, *p<0.05 *vs* NG+ Scrambled siRNA; #*p*<0.05 *vs* HG+ Scrambled siRNA.

High glucose increased expression of Wnt3a mRNA by 144% (Fig 6A, p<0.05) and protein 101% (Fig 6B, p<0.01), β-catenin mRNA by 50% (Fig 6C, p<0.01) and protein by 77% (Fig 6D, p<0.05), and snail mRNA by 51% (Fig 6E, p<0.01) and protein by 107% (Fig 6F, p<0.05) in scrambled siRNA transfected cells. In normal glucose treated cells, PRR siRNA did not cause significant changes in mRNA and protein expressions of Wnt3a, β-catenin or snail in podocytes. In contrast, PRR siRNA treatment caused significant reduction in expression of Wnt3a mRNA by 52% (Fig 6A, p<0.05) and protein by 61% (Fig 6B, p<0.01), β-catenin mRNA by 46% (Fig 6C, p<0.01) and protein by 33% (Fig 6D, p<0.05), and snail mRNA by 34% (Fig 6E, p<0.01) and protein by 49% (Fig 6F, p<0.05) in high glucose treated cells.

### Changes in podocin F-actin and monolayer albumin permeability in response to PRR siRNA

**Figure 7:**
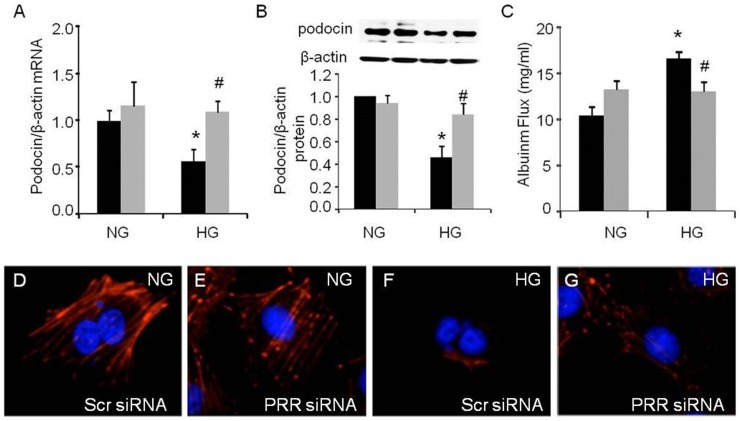
Effect of PRR siRNA on high glucose induced podocyte structure and function. A. Real time PCR analysis of podocin mRNA expression (n = 5); B. Western blot analysis of podocin protein expression (n = 5); C. Analysis of the albumin flux across podocytes monolayer at 2 hours (n = 4); D, E, F and G. Immunofluorescence staining of F-actin shown in red, DAPI shown in blue (n = 5). PRR, (Pro)renin receptor; Normal glucose, 5 mM D-glucose (NG); high glucose, 25 mM D-glucose (HG). Black bar, Scrambled siRNA; Grey bar, PRR siRNA. Data presented as mean ± SEM, *p<0.05 *vs* NG+ Scrambled siRNA; #*p*<0.05 *vs* HG+ Scrambled siRNA.

In normal glucose treated cells, PRR siRNA did not cause significant changes in podocin mRNA or protein expression. In contrast, PRR siRNA significantly increased expressions of podocin mRNA by 98% (Fig 7A, p<0.05) and protein by 83% (Fig 7B, p<0.05) and improved F-actin expression and reorganization (Fig 7D, E, F and G) in high glucose treated podocytes. PRR siRNA significantly attenuated high glucose induced podocytes albumin flux by 21% (Fig 7C, p<0.05).

## Discussion

In the present study, we investigated the role of PRR in high glucose induced podocytes structure and function changes, and explored the mechanisms mediating this pathological process. We demonstrated that PRR mRNA and protein expressions are significantly increased in podocytes exposed to high glucose. This finding is consistent with our previous reports of increased PRR expression in the kidney of a diabetic rat [19] and in high glucose treated mesangial cells [16], [23]. High glucose also induced reduction in podocin and nephrin expression indicating podocytes injury. Downregulation of PRR expression attenuated high glucose induced Wnt3a-β-catenin-snail signaling pathway and improved podocytes structural and functional abnormalities. These results indicate direct involvement of PRR in high glucose induced podocyte structural and functional damage.

In previous studies, non-diabetic mice with podocyte-specific PRR deletion developed nephrotic syndrome and died within 14 days after birth [5], [40]. These reports suggested that under normal conditions, PRR seems to play an important role in the kidney. Recently, we demonstrated increased PRR expression by high glucose [23] and contributes to renal inflammation [18], [23], a response that was attenuated with PRR downregulation. Collectively, these studies suggest that a tight regulation of normal PRR expression is critical for cell structure and function.

In the current study, we hypothesized that high glucose-induced podocyte injury is mediated by enhanced PRR-canonical Wnt signaling pathway, leading to downstream β-catenin accumulation, nuclear translocation and gene transcription [39]. The rationale for this hypothesis is supported by the fact that in non-diabetic state, Wnt-β-catenin activation directly caused podocyte damage through induction of podocyte epithelial-mesenchymal transition (EMT) and down-regulation of nephrin [28], [41], [42]. Pharmacological activation of Wnt-β-catenin signaling pathway caused significant decrease in podocyte adherence and migration, while deletion of β-catenin increased expression of podocytes differentiation markers WT1, podocin, nephrin and synaptopodin [38]. Our data clearly showed increased expression of Wnt3a and β-catenin in podocytes exposed to high glucose, an effect that was attenuated by downregulation of PRR expression. To our knowledge, this is the first report showing that in podocytes, high glucose increases Wnt3a expression via PRR, leading to enhanced Wnt3a-β-catenin signaling pathway.

Next, we demonstrated how activation of Wnt3a-β-catenin signal pathway caused podocytes injury. Snail, one of downstream target genes of Wnt-β-catenin signaling pathway, is an important transcription factor inducing EMT and results in podocytes injury and albuminuria [43]. Previous studies demonstrated that overexpression of snail directly reduced expression of claudin, occludin, E-cadherin, nephrin [44], [45] and induced kidney injury and fibrosis [46]. In our study, we observed that down regulation of PRR inhibited high glucose induced snail mRNA and protein expression. These results suggested that snail gene is one of target effectors of PRR- Wnt3a-β-catenin signaling pathway, which contributes to podocytes injury and dysfunction on exposure to high glucose.

In summary, our results indicated that enhanced PRR expression plays an important role in high glucose induced podocytes structural and functional abnormalities. Wnt3a-β-catenin-snail signaling pathway activation mediates these PRR effects. Decreased expression or activity of PRR in podocytes might be important for reversal of high glucose related podocyte damage. Similarly, reduction in activity of Wnt3a-β-catenin-snail signaling pathway could also help prevents podocyte injury. In conclusion, high glucose causes podocytes injury via enhancement of PRR-Wnt3a-β-catenin-snail signaling pathway.

## Materials and Methods

### Cell culture and treatment

Conditionally immortalized mouse podocyte cell line originally established as previously described [47], and kindly provided by Dr. Mark D. Okusa (Division of Nephrology, University of Virginia Health System, Charlottesville, VA, USA), was cultured as previously described [4], [48]. Cells were cultured on collagen I-coated flasks or plates in RPMI 1640 medium supplemented with recombinant mouse interferon–γ at 33°C. After differentiation at 37°C for 10–14 days without interferon–γ, podocytes were used for the proposed experiments. Podocyte were cultured for 72 hours in medium containing 25 mM D-glucose (high glucose) for experiments groups and 5 mM D-glucose plus 20 Mm L-glucose (normal glucose) for control group.

### PRR siRNA transfection

Transfection of PRR siRNA or scrambled siRNA was performed in six-well plate using the siLentFect lipid reagent (Bio-Rad, Hercules, CA) according to the manufacturer's instructions. One hundred µmol of Accell mouse PRR siRNA - SMARTpool (Thermo Scientific Dharmacon Research Inc, USA, target sequences: 5′-CGAAUAGAUUGAAUUUUCC-3′; 5′-CGGUAUACCUUAAGUUUAU-3′; 5′-UGGUUUAGUAGAGAUAUUA-3′; 5′-GGACCAUCCUUGAGGCAAA-3′) was used for each well. After 6 h incubation in transfection reagent, the cells were then switched to normal medium for overnight recovery and ready for experiment. A scrambled siRNA (QIAGEN, Valencia, CA, target sequences: 5′-AATTCTCCGAACGTGTCACGT-3′), which was confirmed as non-silencing double-stranded RNA, was used as control for siRNA experiments

### Real-time reverse transcription polymerase chain reaction (RT-PCR)

Total RNA from cultured podocytes was extracted using TRIzol reagent (Invitrogen, Carlsbad, CA. USA) according to the protocol as described by the manufacturer. Aliquots of total RNA (1 µg) from each sample were reverse-transcribed into cDNA according to the instructions of the first strand cDNA synthesis kit manufacturer (Bio-Rad, Hercules, CA, USA). Equal amounts of the reverse transcriptional products were subjected to PCR amplification using SYBR Green as the fluorescence indicator on a Bio-Rad iCycler system (Bio-Rad, Hercules, CA, USA). The mRNA levels of target genes were normalized to the β-actin mRNA levels. The primers used in this study were synthesized by Operon (Huntsville, AL, USA) and the sequences were: for PRR, sense TCTCCGAACTGCAAGTGCTA, antisense CTGCAAACTTTTGGAGAGCA; for Wnt3a, sense CGCGCTCTCCAGGCACACTC, antisense TCCGTTGGCCACCACCCTGT; for β-catenin, sense GCCACAGGATTACAAGAAGC, antisense CCACCAGAGTGAAAAGAACG; for snail, sense GCGGAAGATCTTCAACTGCAAATATTGTAAC, antisense GCAGTGGGAGCAGGAGAATGGCTTCTCAC; for nephrin, sense TCTGCCGCCACCTGGTCGTA, antisense ATGGCCCACCTGGGGTCTGG; for podocin, sense CCCTTGTGCTCTGTTGCCGGG, antisense CCAGCCGCTGTCCAGCTTCG and for β-actin, sense TCGCTGCGCTGGTCGTC, antisense GGCCTCGTCACCCACATAGGA.

### Western blot analysis

Western blot analysis was performed as we described previously. In brief, homogenates from cultured podocytes were prepared using sucrose buffer containing protease inhibitor. After boiled for 5 min at 95°C in a 5× loading buffer, 20 µg of total proteins were subjected to SDS-PAGE, transferred onto a PVDF membrane and blocked by solution with dry milk. Then, the membrane was probed with primary antibodies of anti-PRR (1∶1000, Abcam), anti-Wnt3a (1∶1000, Abcam), anti-β-catenin (1∶500, R&D system), anti-snail (1∶500, Novus), anti-nephrin (1∶100, Santa Cruz), anti-podocin (1∶1000, sigma) or anti-β-actin (1∶5000, Santa Cruz) overnight at 4°C followed by incubation with horseradish peroxidase-labeled IgG (1∶5000). The immunoreactive bands were detected by chemiluminescence methods and visualized on Kodak Omat X-ray films. Densitometric analysis of the images obtained from X-ray films was performed using the Image J software (NIH, Bethesda, MD, USA).

### Functional assay of albumin permeability through podocyte monolayer

The permeability of podocyte monolayer to albumin was measured as previously described [4], [49]. Briefly, podocytes were seeded in the upper chambers of 3 µm polycarbonate Transwell filters of a 24-well filtration microplate (Corning, New York, NY). After HG treatment with or without PRR siRNA for 72 h, cells were washed with PBS supplemented with 1 mmol/L MgCl_2_ and 1 mmol/L CaCl_2_ to protect the cadherin-based junctions. The top chamber was filled with 0.15 ml of fresh RPMI 1640 and the bottom chamber with 1 ml of RPMI 1640 supplemented with 40 mg/ml of bovine serum albumin. 2 hours later, the medium from the upper chamber was collected, and the albumin concentration was measured using Bio-Rad protein assay (Bio-Rad).

### PRR Immunofluorescent and immunohistochemical Staining

Cell was seeded in the 8-well chamber slides and treated with HG for 72 hours. For immunofluorescent, podocytes were fixed in 4% PFA for 10 minutes at room temperature and incubated with rabbit anti-PRR (1∶50, Santa Cruz) overnight at 4°C. After washing, the slide was followed by incubation with Alex-594-labeled goat anti-rabbit secondary antibody (1∶500, Invitrogen) and then mounted with DAPI-containing mounting solution and subjected to examinations using a confocal laser scanning microscope (Olympus, Japan). For immunohistochemical staining of PRR, endogenous peroxide activity was suppressed by 0.3% peroxide-methanol solution. Vectastain ABC kit (Vector Laboratories, Burlingame, CA) was used for blocking and color reaction as recommended.

### F-actin staining

Podocytes were seeded in the 8-well chamber slides and treated with HG with or without PRR siRNA for 24 h. After washing with PBS, the podocytes were fixed in 4% paraformaldehyde for 10 min, permeabilized with 0.1% Triton X-100 for 15 min, and blocked with 3% bovine serum albumin for 30 min. F-actin was stained with rhodamine-phalloidin (Invitrogen, Carlsbad, CA, USA) for 20 min at room temperature. After mounting with DAPI-containing mounting solution, the slides were examined by a confocal laser scanning microscope (Olympus, Japan).

### Statistical analysis

All of the values are expressed as mean ± SEM. Significant differences among multiple groups were examined using ANOVA followed by a Student-Newman-Keuls test. χ2 test was used to assess the significance of ratio and percentage data. P<0.05 was considered statistically significant.
